# Abnormal Eosinophils With Large, Distinctly Basophilic Granules (Harlequin Cells) on Peripheral Blood Smear: A Clue for Diagnosing Chronic Myeloid Leukemia

**DOI:** 10.14740/jh2196

**Published:** 2026-04-06

**Authors:** Jennifer Cai, Changjun Yue, Sarah Tomassetti

**Affiliations:** a Harbor-UCLA Medical Center, Torrance, CA 90502, USA; b Charlie Dunlop School of Biological Sciences, University of California Irvine, Irvine, CA, USA; c David Geffen School of Medicine at UCLA, Los Angeles, CA, USA

**Keywords:** Chronic myeloid leukemia, Harlequin cells, Diagnostic screening, acute myeloid leukemia, *CBFB::MYH11*, Primary myelofibrosis, Myeloproliferative neoplasm, Blood smear, Morphologic marker

## Abstract

**Background:**

Chronic myeloid leukemia (CML) often presents with hematologic findings that overlap with reactive leukocytosis and other myeloproliferative neoplasms (MPNs), creating diagnostic uncertainty that may delay targeted therapy or prompt unnecessary molecular testing. Harlequin cells—abnormal eosinophils containing basophilic granules—are well described in acute myeloid leukemia (AML) with *CBFB::MYH11* fusion, but their diagnostic relevance in CML has not been systematically assessed.

**Methods:**

We retrospectively reviewed 177 peripheral blood smears: 53 CML; 30 non-CML MPN and related disorders; 59 AML (including three with *CBFB::MYH11* fusion); 11 eosinophilia; and 24 reactive cytosis cases. Harlequin cells were stringently defined as abnormal eosinophils containing both typical eosinophilic granules and large, distinctly basophilic (not purplish-orange) cytoplasmic granules to exclude reactive mimics.

**Results:**

Harlequin cells were identified in 72% (38 out of 53) of CML cases, a frequency significantly higher than in non-CML MPN (10%, P < 0.01), AML without *CBFB::MYH11* fusion (3.6%, P < 0.01), eosinophilia (0%), and reactive cytosis (0%) groups. They were also observed in 67% (2/3) of AML with *CBFB::MYH11* fusion and in 20% (3/15) of primary myelofibrosis, but were absent in polycythemia vera, essential thrombocythemia, and chronic myelomonocytic leukemia. Strictly defined Harlequin cells were not found in any reactive condition.

**Conclusions:**

In the appropriate clinical context, strictly defined Harlequin cells on routine peripheral blood smears may serve as a sensitive and highly specific morphologic clue for CML. Recognition of this readily accessible feature may facilitate prompt *BCR::ABL1* confirmatory testing, reduce diagnostic ambiguity, and help avoid unnecessary ancillary studies.

## Introduction

Chronic myeloid leukemia (CML) is a myeloproliferative neoplasm (MPN) characterized by leukocytosis with left-shifted neutrophilia and is commonly associated with absolute eosinophilia and absolute basophilia. Atypical presentations, including marked thrombocytosis without leukocytosis, may mimic non-CML MPNs and complicate initial diagnostic impressions. CML is defined by the t(9;22)(q34;q11.2) translocation, which generates the *BCR::ABL1* fusion gene resulting in the production of a BCR::ABL1 fusion protein. The diagnosis is often suspected based on the complete blood count and peripheral blood smear. Once suspected, fluorescence *in situ* hybridization or reverse transcriptase polymerase chain reaction for *BCR::ABL1* can be performed on peripheral blood to confirm the diagnosis. However, the hematological features of CML frequently overlap with those of other conditions such as reactive leukocytosis and other MPNs. This morphologic ambiguity can lead to diagnostic delay, inappropriate laboratory workup, or premature *BCR::ABL1* testing, underscoring the need for simple, cost-effective morphologic clues that can help triage patients for confirmatory molecular testing.

Peripheral blood smear examination, the most accessible and rapid diagnostic tool, may offer such a clue in the form of Harlequin cells—dysplastic eosinophils containing basophilic granules superimposed on typical eosinophilic granules. These cells are well-documented in acute myeloid leukemia (AML) with *CBFB::MYH11* fusion [1–4] and have been sporadically reported in CML [5, 6], suggesting a possible diagnostic association. Yet the literature remains contradictory: some studies propose that Harlequin cells are pathognomonic for specific myeloid neoplasms [3, 7], whereas others describe similar cells in benign bone marrow, arguing against diagnostic specificity [8].

A major source of this discrepancy is the absence of a standardized, rigorous definition of Harlequin cells. Eosinophilic myelocytes in normal or reactive bone marrow often contain both eosinophilic and immature basophilic granules, and mature eosinophils, especially newly formed eosinophils, may rarely exhibit immature granules during heightened eosinopoiesis [9]. Without clear morphologic criteria to distinguish truly abnormal basophilic granules from reactive immature granules, prior studies may have inadvertently included benign mimics, obscuring the diagnostic value of Harlequin cells.

To date, no comprehensive study has systemically evaluated Harlequin cells in CML. The aims of this study were as follows: 1) to establish strict morphologic criteria that distinguish Harlequin cells from normal or reactive eosinophils with immature granules; 2) to determine the prevalence of strictly defined Harlequin cells in CML compared with AML, non-CML MPNs and related disorders, eosinophilia disorders, and reactive cytoses; and 3) to assess the diagnostic utility of these abnormal eosinophils on peripheral blood smears as a morphologic clue to guide *BCR::ABL1* testing and differentiate CML from its clinical mimics.

## Materials and Methods

This research study was conducted retrospectively using archived Wright-Giemsa-stained peripheral blood smears from November 2014 to January 2026 at Harbor-UCLA Medical Center in Southern California. During this time period, a total of 59 patients were diagnosed with CML, 31 with non-CML MPN (including one *SH2B3*-ssociated erythrocytosis), six with chronic myelomonocytic leukemia (CMML), 61 with AML, two with myeloid neoplasm with *PDGFRA* rearrangement, one with lymphocytic variant of hypereosinophilic syndrome (HES), and six with idiopathic HES (iHES), of which peripheral blood smears of 53 CML, 26 non-CML MPN (including one *SH2B3*-ssociated erythrocytosis), four CMML, 59 AML, two myeloid neoplasm with *PDGFRA* rearrangement, one lymphocytic variant of HES, and six iHES cases were available in the archive. Two reactive eosinophilia and 24 reactive cytosis cases were included as controls. Selection of control slides was random, not based on whether or not Harlequin cells were present. Diagnosis and classification of hematological neoplasms was according to the Fifth Edition of the World Health Organization Classification of Hematolymphoid Tumors [10]. One peripheral blood smear was randomly selected from each case. All slides were deidentified and reviewed under a microscope by JC at × 400 and × 1,000 magnifications. The presence or absence of Harlequin cells was recorded. Photomicrographs of suspected Harlequin cells were taken at × 1,000 magnification, and the presence of Harlequin cells was confirmed by CY.

**Figure 1:**
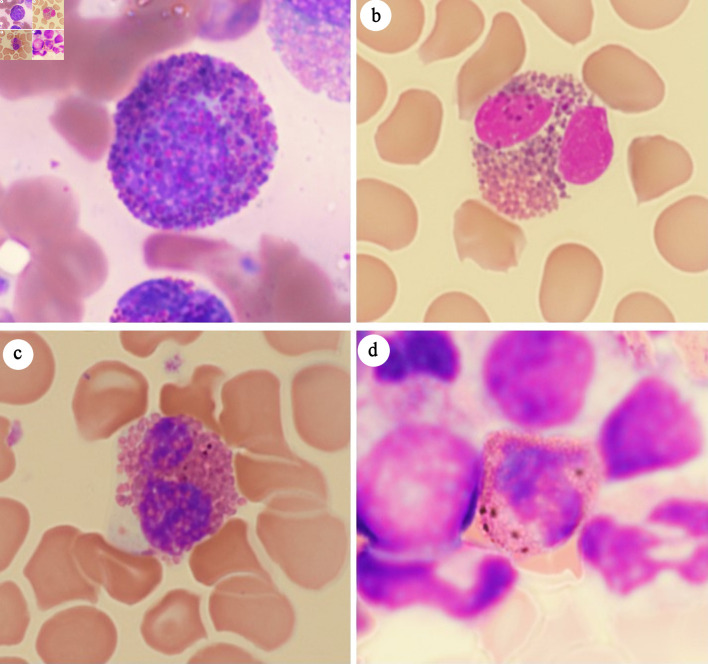
Mimics of Harlequin cells. (a) Eosinophilic myelocyte in reactive bone marrow. (b) Eosinophil with purplish-orange granules. (c) Eosinophil with black granules. (d) Eosinophil with brown granules.

In this study, Harlequin cells were defined as abnormal eosinophils containing both typical eosinophilic granules and large, distinctly basophilic cytoplasmic granules. While normal eosinophilic myelocytes can exhibit immature basophilic granules, these granules usually blend more subtly with the eosinophilic granules in the same cell (Fig. 1a). In contrast, Harlequin cells in myeloid neoplasms display sharp contrasting, often irregular, distinctly basophilic granules that stand out against the eosinophilic background. Purplish-orange granules of normal size and shape (Fig. 1b) and black (Fig. 1c) or brown (Fig. 1d) granules that are refractile when focusing the microscope up and down, were sometimes observed in normal/reactive eosinophils. These granules were not considered as distinctly basophilic granules, and the eosinophils containing these granules did not meet the definition of Harlequin cells in the current study.

### Statistical methods

An overall assessment of the presence or absence of Harlequin cells was determined for each case. A pairwise Fisher’s exact test or Pearson χ^2^ test with Monte-Carlo simulation was used to determine whether the proportion of Harlequin cell presence was different between groups, CML phases, or AML subtypes. Statistical significance was assumed at P ≤ 0.05. The analysis was performed using Kimi K2.5, and the results were checked for accuracy using Microsoft Excel.

Institutional Review Board approval was not required for this study. This study was conducted in compliance with the ethical standards of the responsible institution on human subjects as well as with the Helsinki Declaration.

## Results

The distribution of cases and the incidence of Harlequin cells are summarized in Table 1, and the demographic characteristics of the study population are shown in Table 2. This study included 177 peripheral blood smears, categorized into five groups: CML, non-CML MPN and related disorders, AML, eosinophilia, and reactive cytosis. Age distributions were similar across groups except for the non-CML MPN and related disorders group, which consisted of older patients. There were more males than females in all groups. Among the 53 CML patients, most (n = 44, 83%) were diagnosed in chronic phase, with fewer in accelerated (n = 7, 13%) or in blast phase (n = 2, 4%).

**Table 1:** Summary of Cases

Group	Diagnosis/phase	Number of cases	Incidence of Harlequin cells, n (%) of cases
Chronic myeloid leukemia (CML) (n = 53)	Chronic phase	44	33 (75%)
	Accelerated phase	7	4 (57%)
	Blast phase	2	1 (50%)
	Total	53	38 (72%)
Non-CML myeloproliferative neoplasm and related disorders (n = 30)	Polycythemia vera	5	0 (0%)
	Essential thrombocythemia	5	0 (0%)
	Primary myelofibrosis	15	3 (20%)
	*SH2B3*-associated erythrocytosis	1	0 (0%)
	Chronic myelomonocytic leukemia	4	0 (0%)
	Total	30	3 (10%)
Acute myeloid leukemia (AML) (n = 59)	AML with *CBFB::MYH11* fusion	3	2 (67%)
	AML with *GATA2::MECOM* fusion	2	1 (50%)
	AML with *DEK::DUP214* fusion	1	0 (0%)
	AML with *KMT2A* rearrangement	2	0 (0%)
	AML with *RUNX1::RUNX1T1* fusion	4	0 (0%)
	AML with *NPM1* mutation	10	0 (0%)
	AML, myelodysplasia-related	17	0 (0%)
	AML defined by differentiation	15	1 (7%)
	AML post cytotoxic therapy	5	0 (0%)
	Total	59	4 (7%)
Eosinophilia (n = 11)	Myeloid neoplasm with *PDGFRA* rearrangement	2	0 (0%)
	Lymphocytic variant of hypereosinophilic syndrome	1	0 (0%)
	Idiopathic hypereosinophilic syndrome	6	1 (17%)
	Reactive eosinophilia	2	0 (0%)
	Total	11	1 (9%)
Reactive cytosis (n = 24)	Erythrocytosis/polycythemia	8	0 (0%)
	Thrombocytosis	6	0 (0%)
	Leukemoid reaction	7	0 (0%)
	Leukocytosis + thrombocytosis	3	0 (0%)
	Total	24	0 (0%)

**Table 2:** Demographic Features of Selected Cases

	CML	Non-CML myeloproliferative neoplasm and related disorders	AML	Eosinophilia	Reactive cytosis
Age (years), median (range)	47 (9–75)	65 (20–89)	54 (16–83)	42 (6–89)	55 (20–88)
Sex, n					
Male	33	17	38	6	16
Female	20	13	21	5	8

CML: chronic myeloid leukemia; AML: acute myeloid leukemia.

The non-CML MPN and related disorders group comprised five polycythemia vera (PV), five essential thrombocythemia (ET), 15 primary myelofibrosis (PMF), one *SH2B3*-associated erythrocytosis, and four CMML cases. The AML group (n = 59) included only three cases with *CBFB::MYH11* fusion; the remaining 56 consisted of 22 AML with other recurrent genetic abnormalities, 17 AML with myelodysplasia-related changes, five AML post cytotoxic therapy, and 15 AML defined by differentiation. All 11 eosinophilia cases had an absolute eosinophil count ≥ 1.5 × 10^9^/L and included two myeloid neoplasm with *PDGFRA* rearrangement, one lymphocytic variant of hypereosinophilic syndrome (HES), six idiopathic HES (iHES), and two reactive eosinophilia cases. Because of their significant clinical and laboratory overlap with myeloid neoplasms, eight reactive erythrocytosis/polycythemia, six reactive thrombocytosis, seven leukemoid reaction, and three reactive leukocytosis with thrombocytosis cases were included in the reactive cytosis group.

**Figure 2:**
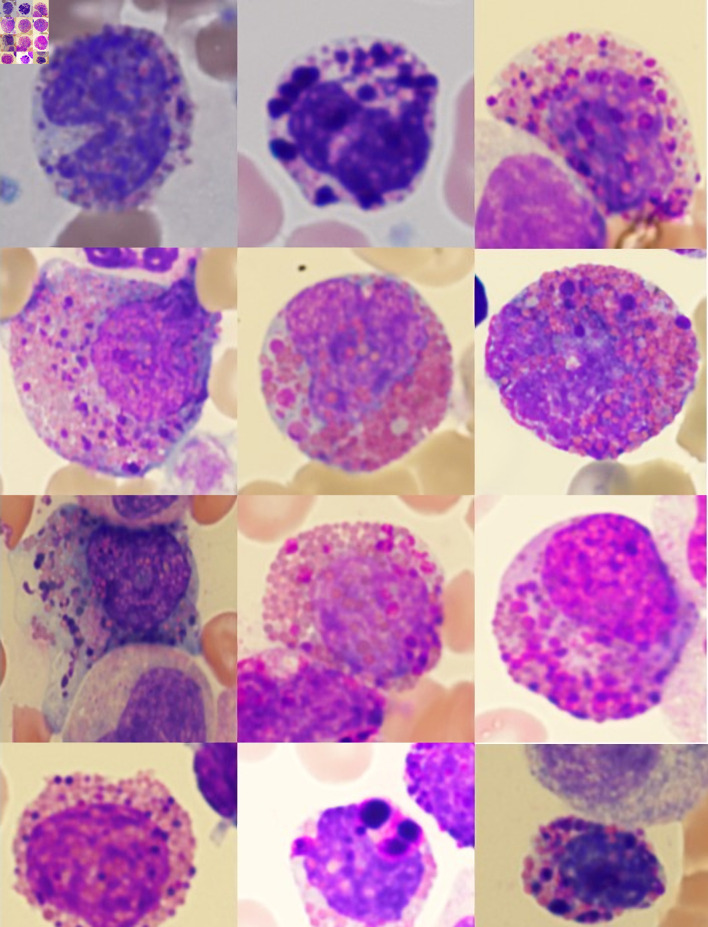
Examples of Harlequin cells in chronic myeloid leukemia.

**Figure 3:**
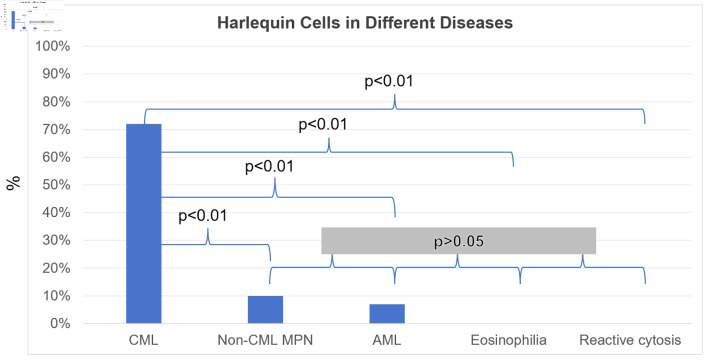
Harlequin cells in chronic myeloid leukemia (CML), acute myeloid leukemia (AML), non-CML myeloproliferative neoplasms and related diseases (non-CML MPN), eosinophilia, and reactive cytosis groups. An overall assessment of the presence or absence of Harlequin cells was determined for each case. Shown are the percentages (%) of cases with Harlequin cells. The positive rate of Harlequin cells in the CML group was significantly higher than that in each of the other groups. However, no significant differences were observed in pairwise comparisons between the other groups.

Using the strict definition, Harlequin cells (Fig. 2) were observed in 72% of the CML blood smears, significantly more frequently than in any other group (Fig. 3). This finding suggests that the strictly defined Harlequin cells could be used as a sensitive and specific morphologic marker for CML in the appropriate clinical context. Harlequin cells were identified in all chronic, accelerated, and blast phases of CML. It seemed more difficult to find Harlequin cells in the accelerated or blast phase, which could be attributed to the reduced eosinophils as blasts increase. However, statistically, the presence of Harlequin cells did not appear to be significantly different across phases (P = 0.51), possibly due to the small number of advanced-phase cases.

**Figure 4:**
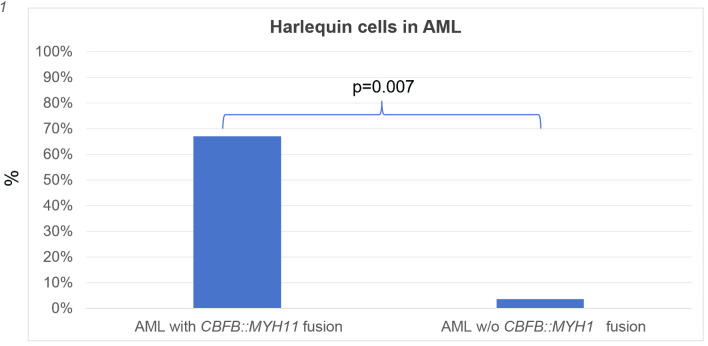
Harlequin cells in acute myeloid leukemia (AML) with or without (w/o) *CBFB::MYH11* fusion. An overall assessment of the presence or absence of Harlequin cells was determined for each case. Shown are the percentages (%) of cases with Harlequin cells. The positive rate of Harlequin cells in AML with *CBFB::MYH11* fusion was significantly higher than that in AML without *CBFB::MYH11* fusion.

Consistent with prior observations, Harlequin cells were detected in two of three blood smears from patients with AML with *CBFB::MYH11* fusion, but were infrequent in other AML cases (2/56, 4%) (Fig. 4). Surprisingly, Harlequin cells were found in three of 15 (20%) PMF cases, but not in PV (0/5), ET (0/5), CMML (0/4), or *SH2B3*-associated erythrocytosis (0/1). However, this difference was not statistically significant (P = 0.24).

**Figure 5:**
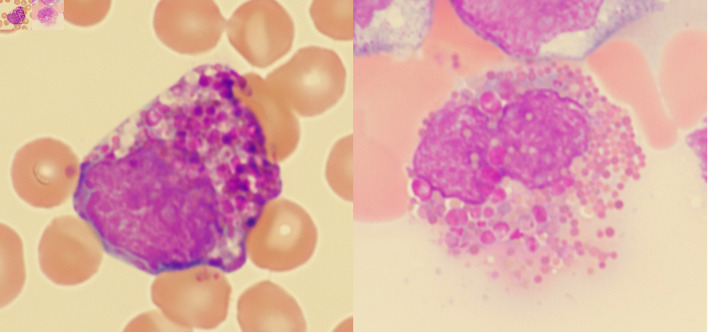
Examples of Harlequin cells in acute myeloid leukemia with *CBFB::MYH11* fusion.

**Figure 6:**
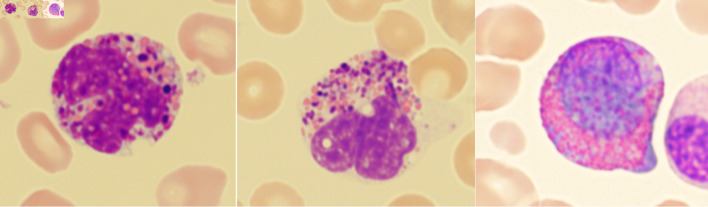
Examples of Harlequin cells in primary myelofibrosis.

**Figure 7:**
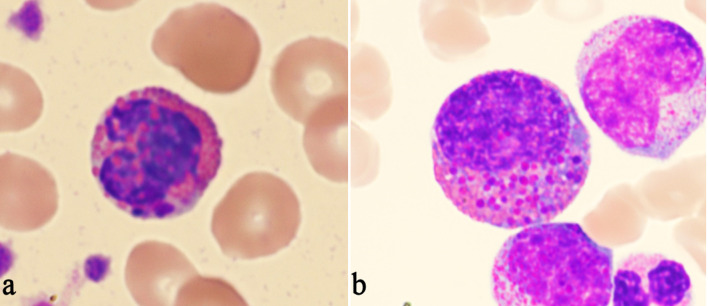
Examples of Harlequin cells in acute myeloid leukemia (AML) without *CBFB::MYH11* fusion. (a) Harlequin cells in AML with *DEK::DUP214* fusion. (b) Harlequin cells in AML defined by differentiation.

Most Harlequin cells observed in AML with *CBFB::MYH11* fusion were eosinophil precursors at the late promyelocyte or myelocyte stage, and the basophilic granules were often abundant, large, irregular, and purple-violet in color (Fig. 5). In CML, Harlequin cells spanned a broader maturation range: some resembled those seen in AML with *CBFB::MYH11* fusion, while others were more mature eosinophils containing deeply basophilic granules of variable size (Fig. 2). Harlequin cells in PMF were similar to those in CML (Fig. 6). Only one case of AML with *DEK::DUP214* fusion and one case of AML defined by differentiation showed Harlequin cells in the peripheral blood smears (Fig. 7).

**Figure 8:**
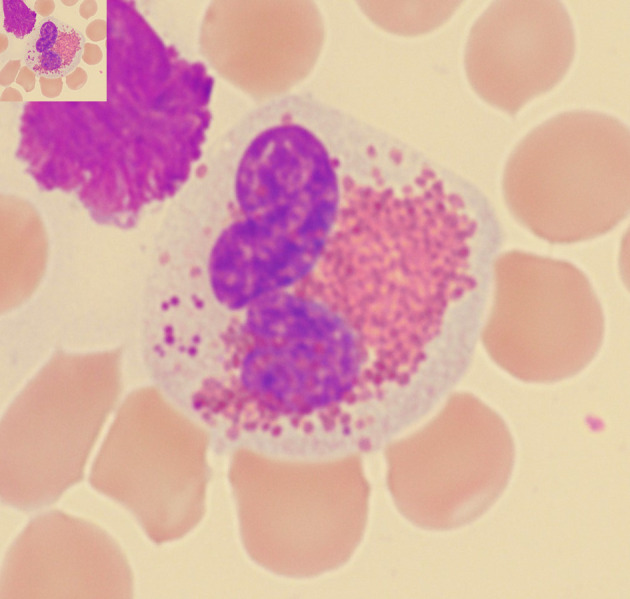
Small purple granules in one eosinophil in a case of idiopathic hypereosinophilic syndrome.

Virtually, no Harlequin cells were found in eosinophilia group or reactive cytosis group except that a few small purple granules were observed in one eosinophil in a case of iHES (Fig. 8).

## Discussion

The present study demonstrates that strictly defined Harlequin cells represent a practical and informative diagnostic biomarker for CML, offering a rare combination of high sensitivity (72%), exceptional specificity (94–100% against mimics), and immediate availability at the point of care. By applying rigorous morphologic criteria that exclude immature granules in normal or reactive eosinophils, we show that these abnormal eosinophils are not merely morphologic curiosities but reproducible indicators of myeloid neoplasm that can be readily identified on routine Wright-Giemsa-stained peripheral blood smears. This finding is particularly valuable in resource-limited settings where immediate *BCR::ABL1* testing may not be accessible.

The mechanisms underlying Harlequin cell formation remain poorly understood. Basophilic granules within eosinophils may arise from genetic abnormalities such as *CBFB::MYH11*, aberrant eosinophil-basophil lineage commitment from a shared progenitor [11], or broader neoplastic transformation. Whether these mechanisms differ across disease entities or reflect a common pathway is unknown. The striking restriction of Harlequin cells to CML (72%), *CBFB::MYH11*-positive AML (67%), and a subset of PMF (20%)—with complete absence in reactive conditions, PV, ET, and CMML—suggests that these cells may represent a specific morphologic signature of dysregulated eosinophil-basophil differentiation driven by genetic (e.g., *BCR::ABL1*, *CBFB::MYH11*) and/or molecular lesions.

The observation that PMF demonstrated a 20% positivity rate, while other non-CML MPNs showed none, raises the intriguing possibility that Harlequin cells may help distinguish PMF from PV and ET—an area of frequent diagnostic challenge in clinical practice. However, given the limited sample sizes for PMF, PV, and ET, this finding should be interpreted cautiously and validated in larger cohorts before clinical application.

Our findings also help reconcile conflicting reports regarding Harlequin cell specificity. Studies describing their ubiquitous presence in benign bone marrows [8] likely included normal eosinophilic myelocytes. By establishing strict morphologic criteria and excluding reactive mimics, we demonstrate that definition is critical: when properly identified, Harlequin cells are not benign variations but reliable neoplastic markers.

The 67% prevalence of Harlequin cells in *CBFB::MYH11*-positive AML confirms prior observations, supporting their use as an adjunctive morphologic clue for this cytogenetic entity. In the AML diagnostic workup, their presence should prompt targeted *CBFB::MYH11* testing, potentially expediting risk stratification and therapeutic decision-making.

As a single-center retrospective study, our findings require external validation. Reviewing only one peripheral blood smear per case, while reflecting real-world practice where pathologists typically examining one or two slides, may underestimate Harlequin cell detection in cases with eosinopenia. Future prospective studies should determine the minimal number of eosinophils required for reliable Harlequin cell detection and further explore the potential diagnostic utility in PMF and other non-CML MPNs. Additionally, digital pathology and artificial intelligence tools may help standardize Harlequin cell recognition, reduce interobserver variability, and facilitate broader adoption.

### Conclusions

Strictly defined Harlequin cells on peripheral blood smears serve as a rapid, cost-effective, and reliable screening tool for CML, combining high sensitivity with excellent specificity against reactive and neoplastic mimics. In the appropriate clinical and laboratory context, their identification can guide appropriate *BCR::ABL1* testing, reducing diagnostic delays and avoiding unnecessary molecular studies. Harlequin cells also provide a valuable morphologic clue for *CBFB::MYH11* fusion in AML and may offer preliminary discriminatory value between PMF and other non-CML MPNs. In an era dominated by advanced molecular diagnostics, this simple microscopic finding underscores the enduring diagnostic power of careful morphologic evaluation to deliver immediate, actionable insights at the point of care.

## Data Availability

The data used to support the findings of this study are available from the corresponding author upon request.
